# Associations of fasting glucose and glycated hemoglobin with vitamin D levels according to diabetes mellitus status in Korean adults

**DOI:** 10.4178/epih.e2022025

**Published:** 2022-02-21

**Authors:** Yerin Hwang, Jiyoung Jang, Myung-Hee Shin

**Affiliations:** Department of Social and Preventive Medicine, Sungkyunkwan University School of Medicine, Suwon, Korea

**Keywords:** Diabetes mellitus, Blood glucose, Glycated hemoglobin A1c, Vitamin D, Korea

## Abstract

**OBJECTIVES:**

According to previous studies, vitamin D deficiency might increase the risk of type 2 diabetes mellitus (DM). However, few studies have examined whether vitamin D continues to affect glucose control after DM diagnosis. Therefore, we examined the association between vitamin D and glucose levels in individuals with and without DM.

**METHODS:**

We analyzed data for 32,943 adults aged 19 years and older from the 2008 to 2014 Korea National Health and Nutrition Examination Survey. Patients were classified into 3 groups according to the 25-hydroxyvitamin D concentration. DM was defined as a fasting glucose level ≥126 mg/dL, current use of DM medications or insulin injections, or a self-reported diagnosis of DM by a doctor.

**RESULTS:**

In male DM patients, the hemoglobin A1c (HbA1c) level increased significantly as vitamin D levels became severely deficient. In male and postmenopausal female with abnormal HbA1c, those with severe vitamin D deficiency had significantly higher HbA1c levels (p for trend=0.004 and 0.022 for male and postmenopausal female, respectively). Significant differences were found between participants with normal and abnormal HbA1c levels in both male and female. However, regardless of sex or menopausal status, there was no significant association between vitamin D and fasting glucose in any of the fasting glucose subgroups.

**CONCLUSIONS:**

Male and female with abnormal HbA1c levels showed markedly elevated blood glucose when they also had vitamin D deficiency. A more distinct difference was observed in the HbA1c subgroups than in the fasting glucose subgroups.

## INTRODUCTION

Vitamin D is abundant in several types of fish and egg yolks [1], but only a small amount of vitamin D intake occurs via food consumption; instead, most is produced by the body upon exposure to sunlight [2]. There is increasing interest in vitamin D because vitamin D deficiency has become common both in Korea and in other countries [3]. Low vitamin D levels are associated with mental disorders, cancer, and cardiovascular disease [4-7]. Some research has also shown that vitamin D affects insulin sensitivity and insulin secretion, as insulin secretion was reduced in vitamin D-deficient rats, and their insulin levels increased after they received an injection of vitamin D [8]. Because vitamin D affects insulin secretion and insulin sensitivity, which are important factors in the development of diabetes mellitus (DM), it is possible that vitamin D levels are linked to the development of DM.

DM is a chronic disease caused by defects in insulin secretion or regulation [9], and the increasing prevalence of DM has become a social and health problem worldwide [10]. According to the Korean Diabetes Association, the prevalence of DM among Korean adults older than 30 years in 2018 was 13.8%, which represents an increased prevalence over the past 7 years [11]. Furthermore, the International Diabetes Federation reported that DM patients numbered about 463 million in 2019, but that number was expected to increase to about 700 million by 2045 [12]. DM causes complications such as stroke, cardiovascular disease, retinopathy, and nephropathy, and it increases patients’ mortality rate [13,14]. In addition, DM patients with a longer illness duration experience greater morbidity and DM-related mortality rates than those with a shorter duration [15,16]. The ongoing management of DM produces an ever-growing burden of medical expenses, such that low-income groups often show poor medical compliance [17,18].

Previous studies investigating the association between vitamin D and the development of DM have reported that individuals with vitamin D deficiency had an increased prevalence of type 2 DM [19,20]. Another study reported that supplementation with vitamin D and calcium decreased fasting glucose in individuals with impaired glucose tolerance [21], implying that vitamin D can help prevent DM. According to a recent meta-analysis of DM patients, vitamin D supplementation decreased insulin resistance compared with a placebo group, but it had no effect on fasting glucose or hemoglobin A1c (HbA1c) [22]. Few studies have investigated the association between vitamin D levels and glucose control among Korean DM patients.

Although many studies have sought to establish an association between vitamin D levels and DM, only a few studies have included Korean patients with DM. Furthermore, the mechanism by which vitamin D affects glucose control in DM patients is unclear. Therefore, in this study, we examined differences in the associations between vitamin D levels and glucose in individuals with and without DM using data from the Korea National Health and Nutrition Examination Survey (KNHANES) from 2008 to 2014.

## MATERIALS AND METHODS

### Study population

**Figure 1.**
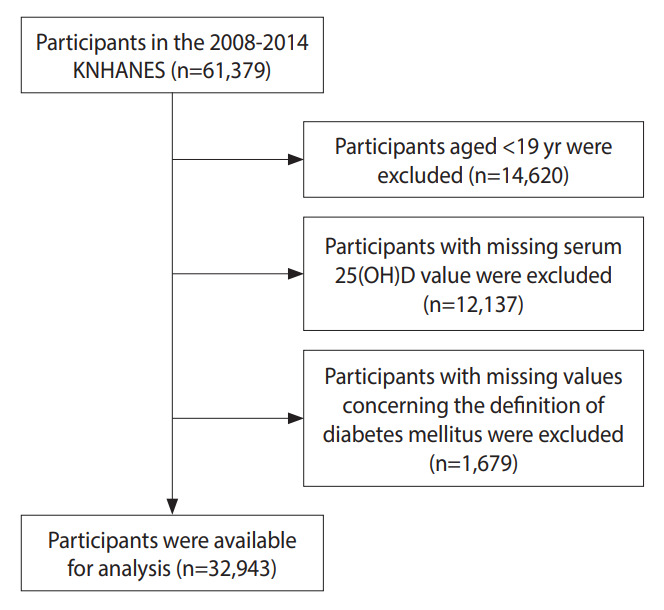
Flow chart of study participant selection from the 2008 to 2014 Korea National Health and Nutrition Examination Survey (KNHANES). 25(OH)D, 25-hydroxyvitamin D.

This study used data from the KNHANES, a study initiated in 1998; the data from KNHANES VIII (2019-) remain in progress. We used data from the second year of the KNHANES IV (2008) to the second year of the KNHANES VI (2014), which included 25-hydroxyvitamin D (25[OH]D) concentration results for participants. Among the 61,379 participants, we excluded those younger than 19 years (n=14,620), those who had missing serum 25(OH) D values (n=12,137), and those who had missing values for variables related to the definition of DM (n=1,679). Therefore, we analyzed data from 32,943 participants (Figure 1).

### Demographic and lifestyle information

The variables analyzed as general characteristics of the study participants were age, sex, total energy intake, education level, household income level, smoking status, obesity, and physical activity. Total energy intake was calculated as the total daily calories consumed by each person as reported using the 24-hour recall method. Participants were divided into 4 categories according to their education level (< elementary school graduates, middle school graduates, high school graduates, ≥ college graduates), and household income was divided into quartiles according to the equivalized income (total household income divided by the square root of the number of household members). Participants were categorized according to their smoking status as current smokers, previous smokers, or never smokers. Obesity was stratified into 3 categories after calculating the body mass index (BMI) from height and weight. According to the World Health Organization’s criteria for the Asian population, the BMI cut-off values for underweight and obese individuals are less than 18.5 kg/m^2^ and at least 25.0 kg/m^2^, respectively [23]. Participants who performed moderate physical activity for at least 30 min/day on 5 day/wk or vigorous physical activity for at least 20 min/day on 3 day/wk were considered to have a high exercise level, while the others were classified as engaging in a low level of exercise.

### Measurement of vitamin D levels

The serum 25(OH)D concentration was measured by radioimmunoassay using a 25(OH)D 125I radioimmunoprecipitation assay kit and a 1470 WIZARD gamma-counter (PerkinElmer, Waltham, MA, USA). Three categories of 25(OH)D levels were defined: severe deficiency (< 10.0 ng/mL), deficiency (10.0-< 20.0 ng/mL), and sufficiency (≥ 20.0 ng/mL) [24,25].

### Definition of diabetes mellitus and blood glucose status

All study participants had blood testing results from samples taken following a fast of longer than 8 hours. DM patients were defined as those with a fasting glucose level ≥ 126 mg/dL, current use of DM medication or insulin injections, or a self-reported diagnosis of DM by a doctor. Participants were subgrouped using an HbA1c concentration of 6.5% and a fasting glucose level of 126 mg/dL for the analyses of blood glucose status [26].

### Statistical analysis

All statistical analyses involved a complex survey design, sampling weights, and stratified and clustered sampling. The chi-square test was applied to categorical variables to estimate the frequencies and percentages of participants’ characteristics according to their vitamin D levels. Differences in continuous variables among the vitamin D level subgroups were assessed by one-way analysis of variance to estimate the means and standard errors, followed by the Tukey multiple comparison test.

To determine the associations of vitamin D levels with DM, we ran a multiple linear regression analysis stratified according to the prevalence of DM and glucose levels. Age, sex, total energy intake, education level, smoking status, obesity, and physical activity were considered as confounding factors. We also conducted interaction tests to confirm whether differences in the associations with normal and abnormal glucose levels were significant. The dependent variables were fasting glucose and HbA1c, which were both analyzed as continuous variables. DM status and categorical variables for fasting glucose and HbA1c (defined using cut-off levels) were used as interaction variables. For normality, fasting blood glucose and HbA1c levels were analyzed by log transformation, and the relevance was compared according to sex and menopausal status. All analyses were conducted using SAS version 9.4 (SAS Institute Inc., NC, USA), and significance was defined as a 2-tailed p-value less than 0.05.

### Ethics statement

The use of the original data from the KNHANES in this study adheres to the personal information protection and statistics law, and it provides the only data that cannot be estimated from the survey data. The researcher applied for the required information on the Korea Centers for Disease Control and Prevention website before starting the study.

## RESULTS

Among the 32,943 study participants, the mean age was 44.15 years, and the mean serum 25(OH)D concentration was 17.53 ng/mL. Participants’ general characteristics according to their vitamin D levels are shown in Table 1. Overall, 2,650 (8.6%) participants were classified as having severe vitamin D deficiency, 19,633 (61.6%) were classified as having vitamin D deficiency, and 10,660 (29.8%) had sufficient vitamin D levels. Participants in the severe deficiency and deficiency subgroups had significantly lower ages, total energy intake, proportion of current smokers, and rates of obesity and physical activity than those in the sufficient subgroup (p<0.001). In contrast, participants with severe vitamin D deficiency had a significantly higher education level than those in the other subgroups, and this group had the highest proportion of female (p<0.001). The prevalence of DM was highest in the sufficient subgroup (p<0.001).

Table 2 presents the associations between vitamin D levels and fasting glucose after stratification by DM status. Among DM patients, both fasting glucose and HbA1c levels were significantly higher in the severe vitamin D deficiency subgroup than in the sufficient subgroup (p for trend=0.012 and < 0.001 for fasting glucose and HbA1c, respectively), whereas these variables were not correlated in the participants without DM. Additionally, in the analyses stratified by the cut-off value for fasting glucose (126 mg/dL), we found that vitamin D levels showed significant inverse associations with fasting glucose and HbA1c in participants with abnormal fasting glucose levels (p for trend=0.032 and < 0.001 for fasting glucose and HbA1c, respectively), but no significant association was found in individuals with normal fasting glucose levels. In the analyses stratified by the HbA1c cut-off value (6.5%), we found a significant inverse association between vitamin D and glucose control in the abnormal subgroup (p for trend=0.012 and < 0.001 for fasting glucose and HbA1c, respectively), and a significant association between vitamin D and fasting glucose in the normal HbA1c level subgroup (p for trend=0.034).

To confirm whether the associations with vitamin D differed according to sex or menopausal status, we conducted stratified analyses. In male, the HbA1c level increased significantly as vitamin D became severely deficient in DM patients and in the abnormal fasting glucose and abnormal HbA1c subgroups (p for trend < 0.001 for DM patients, the abnormal fasting glucose subgroup, and the abnormal HbA1c subgroup, respectively), whereas there was no significant association between vitamin D and fasting glucose in any normal subgroup (Table 3). In female, there was a significant association between vitamin D levels and fasting glucose and HbA1c in the abnormal HbA1c subgroup (p for trend=0.004 and 0.005 for fasting glucose and HbA1c, respectively) (Table 3). Table 4 shows that before menopause, there was a significant association between vitamin D and glucose control in female with normal HbA1c levels, but there were no significant differences between the normal and abnormal HbA1c subgroups (p for interaction=0.389). After menopause, however, fasting glucose levels increased significantly in DM patients and those in the abnormal HbA1c subgroup. HbA1c levels were also significantly higher in the abnormal HbA1c level subgroup (Table 4). Regardless of sex or menopausal status, there was no significant association between vitamin D and glucose levels in individuals without DM.

In logistic regression, we found no significant association between vitamin D levels and DM risk before menopause (Supplementary Material 1). Even when stratified by supplements or daily calcium intake to confirm the independent association between calcium intake and vitamin D, the association between vitamin D and DM risk was significant only in DM patients (Supplementary Material 2).

## DISCUSSION

Male and female with abnormal HbA1c showed markedly elevated blood glucose levels when they also had vitamin D deficiency. Furthermore, there was a more distinct difference in the HbA1c subgroups than in the fasting glucose subgroups.

In humans, 7-dehydrocholesterol present in the skin is converted to pre-vitamin D3 by ultraviolet rays and then into vitamin D3 deeper in the body [27]. Vitamin D3 is combined with vitamin D–binding protein synthesized in the liver to become 25(OH)D, which is hydroxylated by 1α-hydroxylase in the kidney to become 1,25-dihydroxy vitamin D (1,25[OH]_2_D_3_) [28]. Vitamin D receptors and vitamin D–dependent calcium-binding proteins are present in pancreatic β-cells and can affect insulin secretion and insulin sensitivity [29]. Vitamin D affects insulin action by regulating extracellular calcium (Ca^2+^), which maintains the influx of normal calcium through the cell membrane, and controlling the appropriate intracellular cytoplasmic Ca^2+^ pool [30]. Therefore, when vitamin D is insufficient, parathyroid hormone (PTH) can increase, which elevates the concentration of free intracellular calcium in insulin-responsive tissues, including skeletal muscle and adipose tissue. This can deform the balance between the intracellular and extracellular β-cell Ca^2+^ pools, disturbing normal insulin secretion in response to glucose [31]. Furthermore, PTH is associated with insulin resistance, and one study showed that PTH decreased insulin-stimulated glucose absorption in rat adipocytes [32]. In other words, PTH can cause insulin resistance by decreasing the amount of glucose transporter (GLUT)1 and GLUT4 in cell membranes [33]. Thus, PTH promotes insulin resistance by reducing glucose absorption in the liver, muscle, and adipose tissue [34]. However, the mechanism involved has not yet been clarified.

A meta-analysis of recent studies confirmed an association between vitamin D supplementation and fasting glucose levels in individuals without DM, but vitamin D did not generally show a significant association with fasting glucose levels; only the subgroup of participants with a BMI < 25.0g/m^2^ and the subgroup with a 25(OH)D concentration in the range of 20.0-30.0 ng/mL showed a significant decrease in fasting glucose [35]. This is consistent with our results, which show no significant association between vitamin D and fasting glucose or HbA1c in participants without DM. In contrast, a meta-analysis of observational studies found an association between vitamin D deficiency and type 2 DM and reported a significant inverse association between vitamin D and glucose levels in both DM patients and individuals without DM [36]. However, most of the studies included in that meta-analysis were conducted in other countries, and few studies have focused on Koreans.

Many studies have investigated the role of vitamin D supplementation in glucose control in DM patients, but their results have been inconsistent. According to a recent meta-analysis of type 2 DM patients, their fasting glucose, HbA1c, and homeostatic model assessment for insulin resistance (HOMA-IR) scores all decreased significantly with vitamin D supplementation of at least 4,000 IU/day [37]. However, another meta-analysis published in the same year reported finding insufficient evidence to indicate that glucose metabolism or insulin improved in DM patients following supplementation with vitamin D [38]. In one of the studies included in the latter meta-analysis, a significant decrease in both glucose and HOMA-IR score was observed in patients treated with 1,000 IU of vitamin D and 300 mg of calcium [39]. However, most studies included in both meta-analyses of DM patients were conducted outside Korea; a study of Koreans did not demonstrate significant changes following vitamin D and calcium supplementation [40]. One reason for that result is that only about 44% of those who received vitamin D and calcium reached target vitamin D levels.

Because DM is a chronic disease with a high risk of complications, continuous blood glucose control is important [26]. However, according to a 2020 DM fact sheet published by the Korean Diabetes Association, only about 28.3% of DM patients in Korea control their HbA1c concentration to less than 6.5%, and 19.1% of patients had an HbA1c concentration greater than 8.0% [11]. An international cross-sectional study confirmed an inverse association between HbA1c and the serum 25(OH)D concentration in DM patients after adjusting for DM severity [41]. In addition, a study on the association between HbA1c control and vitamin D deficiency in DM patients in Pakistan reported greater vitamin D deficiencies in DM patients whose HbA1c concentrations were 7.0% or higher [42]. Those previous studies suggest that vitamin D plays an important role in controlling blood glucose in DM patients.

Therefore, we conducted stratified analyses according to DM status and glucose levels to determine whether the role of vitamin D differs between DM prevention and progression. We found a significant association between vitamin D and glucose control in male and postmenopausal female with abnormal HbA1c levels. Therefore, we infer that vitamin D is more valuable as a prognostic factor among DM patients whose glucose is high than as a preventive factor among people without DM. Premenopausal female with normal HbA1c levels showed a significant association between vitamin D and glucose control, indicating that estrogen may be associated with glucose control in that population. A previous study showed that most postmenopausal female experience a dramatic increase in insulin resistance [43]. Estrogen might regulate insulin through insulin-sensitive tissues or by regulating factors such as oxidative stress that contribute to insulin resistance [44].

This study has some limitations and strengths that should be noted. First, this was a cross-sectional study, so we cannot comment on the causal relationship between vitamin D levels and DM status. Second, we did not consider the exposure time and season, which could affect vitamin D levels. However, to address those factors as much as possible, we did perform our statistical analyses after adjusting for confounders. Third, we could not classify or analyze the type of DM because the KNHANES does not distinguish between type 1 DM and type 2 DM. However, because type 1 DM occurs mostly in childhood and has a low prevalence [45], it can be assumed that most DM patients included in this study had type 2 DM. Furthermore, although the KNHANES data do not include information about the level of dietary vitamin D, the serum 25(OH)D level used in this study encompasses both the vitamin D concentration produced by synthesis in the skin and dietary vitamin D intake [46]. This study has the strengths of analyzing a representative sample of Korean adults using KNHANES data and investigating the relationship between vitamin D and blood glucose in participants with and without DM, which allowed us to evaluate the differences in associations between vitamin D and blood glucose control according to DM status. Since our results confirm the relationship between vitamin D and blood glucose, and most of the Koreans in our study population had insufficient vitamin D levels, this work could be used to prepare management guidelines for blood glucose control.

In conclusion, we found a difference in the association between vitamin D and glucose control according to DM status in Korean adults, which is a novel contribution to the scientific literature. Guidelines are needed for managing glucose control in DM patients that reflect the results of this research performed among Koreans. Additional large-scale longitudinal studies should be conducted to clarify the causal relationships underlying this association.

## Figures and Tables

**Table 1.** Characteristics of subjects by vitamin D level

Characteristics		Sufficiency (≥20.0 ng/mL)	Deficiency (10.0-<20.0 ng/mL)	Severe deficiency (<10.0 ng/mL)	p-value^1^
Total (n)		10,660	19,633	2,650	
25(OH)D (ng/dL)		25.35±0.08	15.03±0.04	8.42±0.04	<0.001
Age (yr)^2^		48.59±0.26^a^	42.53±0.17^b^	40.36±0.39^c^	<0.001
Sex					<0.001
	Male	5,721 (60.8)	7,818 (48.0)	727 (35.5)	
	Female	4,939 (39.2)	11,815 (52.0)	1,923 (64.5)	
Total energy intake (kcal/day)^2^		2,108.63±13.79^a^	2,013.87±10.03^b^	1,843.22±22.04^c^	<0.001
Education level					<0.001
	≤Elementary school graduation	3,429 (23.4)	4,163 (14.6)	527 (13.7)	
	Middle school graduation	1,438 (12.7)	1,904 (8.8)	231 (7.6)	
	High school graduation	3,237 (36.2)	7,175 (41.4)	1,035 (44.8)	
	≥College graduation	2,526 (27.6)	6,362 (35.2)	851 (33.9)	
Household income level					<0.001
	Low	2,422 (17.2)	3,173 (13.2)	456 (15.7)	
	Low-middle	2,674 (26.1)	4,885 (25.9)	713 (27.4)	
	High-middle	2,672 (27.7)	5,593 (30.5)	752 (30.0)	
	High	2,772 (28.9)	5,743 (30.4)	682 (26.8)	
Smoking status					<0.001
	Never	5,520 (47.0)	12,024 (55.4)	1,809 (62.1)	
	Former	1,510 (15.6)	2,325 (12.8)	212 (8.6)	
	Current	3,608 (37.5)	5,250 (31.8)	622 (29.4)	
Obesity (kg/m²)					<0.001
	Underweight (<18.5)	386 (3.5)	877 (4.8)	216 (8.9)	
	Normal (18.5-<25.0)	6,728 (63.2)	12,449 (63.4)	1,717 (65.4)	
	Obese (≥25.0 kg/m²)	3,496 (33.3)	6,192 (31.7)	678 (25.7)	
Physical activity					<0.001
	No	7,749 (71.4)	15,076 (75.6)	2,109 (77.4)	
	Yes	2,871 (28.6)	4,498 (24.4)	530 (22.6)	
Fasting glucose level (mg/dL)^2^		97.88±0.25^a^	96.14±0.19^b^	95.68±0.61^b^	<0.001
HbA1c (%)^2^		5.90±0.02^a^	5.77±0.01^b^	5.80±0.04^a,b^	<0.001
Diabetes mellitus					<0.001
	No	9,456 (90.2)	17,832 (92.6)	2,378 (91.9)	
	Yes	1,204 (9.8)	1,801 (7.4)	272 (8.12)	

Values are presented as mean±standard error or number (%). 25(OH)D, 25-hydroxy vitamin D; HbA1c, hemoglobin A1c.

1 From the chi-square test for categorical variables and from generalized linear regression analysis for continuous variables.

2 Different letters represent statistically significant differences by the Tukey multiple comparison test.

**Table 2.** Associations of vitamin D levels with log(fasting glucose) and log(HbA1c) by diabetes status and glucose level

Vitamin D levels		Diabetes	Non-diabetes	p for interaction	Fasting glucose		p for interaction	HbA1c		p for interaction
					Abnormal	Normal		Abnormal	Normal	
Log (fasting glucose)				<0.001			0.003			0.003
	Sufficiency (≥20 ng/mL)	Reference	Reference		Reference	Reference		Reference	Reference	
	Deficiency (10-<20 ng/mL)	0.0297^*^	0.0014		0.0172	0.0007		0.0315^*^	0.0039	
	Severe deficiency (<10 ng/mL)	0.0466	0.0012		0.0571	0.0007		0.0633	0.0105^*^	
	p for trend	0.012	0.472		0.032	0.712		0.012	0.034	
Log (HbA1c)				<0.001						<0.001
	Sufficiency (≥20 ng/mL)	Reference	Reference		Reference	Reference	<0.001	Reference	Reference	
	Deficiency(10-<20 ng/mL)	0.0291^**^	0.0007		0.0297^**^	-0.0008		0.0251^**^	0.0010	
	Severe deficiency (<10 ng/mL)	0.0645^**^	0.0009		0.0795^**^	0.0020		0.0689^**^	0.0008	
	p for trend	<0.001	0.719		<0.001	0.794		<0.001	0.651	

Values are presented as β coefficient. Adjusted for age, sex, total energy intake, education level, smoking status, obesity, and physical activity. HbA1c, hemoglobin A1c.

* p<0.05,

** p<0.01.

**Table 3.** Association of vitamin D with log(fasting glucose) and log(HbA1c) by diabetes status and glucose level by sex

Vitamin D levels			Diabetes	Non-diabetes	p for interaction	Fasting glucose		p for interaction	HbA1c		p for interaction
						Abnormal	Normal		Abnormal	Normal	
Male											
	Log (fasting glucose)				0.042			0.066			0.161
		Sufficiency (≥20 ng/mL)	Reference	Reference		Reference	Reference		Reference	Reference	
		Deficiency (10-<20 ng/mL)	0.0287	0.0022		0.0142	0.0016		0.0353	0.0029	
		Severe deficiency (<10 ng/mL)	0.0535	-0.0006		0.0890	-0.0009		0.0329	0.0084	
		P for trend	0.092	0.623		0.086	0.759		0.185	0.299	
	Log (HbA1c)				<0.001			<0.001			0.003
		Sufficiency (≥20 ng/mL)	Reference	Reference		Reference	Reference		Reference	Reference	
		Deficiency (10-<20 ng/mL)	0.0365^**^	0.0003		0.0392^**^	-0.0007		0.0344^**^	0.0011	
		Severe deficiency (<10 ng/mL)	0.1063^**^	-0.0012		0.1368^**^	0.0021		0.0756	-0.0035	
		P for trend	<0.001	0.926		<0.001	0.923		0.004	0.831	
Female											
	Log (fasting glucose)				0.010			0.057			0.003
		Sufficiency (≥20 ng/mL)	Reference	Reference		Reference	Reference		Reference	Reference	
		Deficiency (10-<20 ng/mL)	0.0357	0.0012		0.0263	0.0001		0.0409	0.0065	
		Severe deficiency (<10 ng/mL)	0.0432	0.0023		0.0435	0.0016		0.0980^**^	0.0138^**^	
		p for trend	0.079	0.437		0.118	0.678		0.004	0.008	
	Log (HbA1c)				0.011			0.080			0.001
		Sufficiency (≥20 ng/mL)	Reference	Reference		Reference	Reference		Reference	Reference	
		Deficiency (10-<20 ng/mL)	0.0187	0.0006		0.0160	-0.0013		0.0183	0.0007	
		Severe deficiency (<10 ng/mL)	0.0307	0.0024		0.0382	0.0025		0.0641^**^	0.0036	
		p for trend	0.111	0.535		0.161	0.713		0.005	0.326	

Values are presented as β coefficients. Adjusted for age, total energy intake, education level, smoking status, obesity, and physical activity. HbA1c, hemoglobin A1c.

** p<0.01.

**Table 4.** Associations of vitamin D with log(fasting glucose) and log(HbA1c) by diabetes status and glucose level in menopausal status female^1^

Vitamin D levels			Diabetes	Non-diabetes	p for interaction	Fasting glucose		p for interaction	HbA1c		p for interaction
						Abnormal	Normal		Abnormal	Normal	
Berore menopaus											
	Log (fasting glucose)				0.921			0.564			0.389
		Sufficiency (≥20 ng/mL)	Reference	Reference		Reference	Reference		Reference	Reference	
		Deficiency (10-<20 ng/mL)	0.0163	0.0022		-0.0190	0.0029		-0.0100	0.0128^**^	
		Severe deficiency (<10 ng/mL)	0.0311	0.0040		0.0168	0.0045		0.0830	0.0183^**^	
		p for trend	0.736	0.293		0.903	0.224		0.531	0.005	
	Log (HbA1c)				0.357			0.519			0.048
		Sufficiency (≥20 ng/mL)	Reference	Reference		Reference	Reference		Reference	Reference	
		Deficiency (10 -<20 ng/mL)	0.0195	0.0004		-0.0009	0.0016		0.0312	0.0011	
		Severe deficiency (<10 ng/mL)	0.0608	0.0010		0.0446	0.0026		0.1126^*^	0.0026	
		p for trend	0.333	0.825		0.551	0.575		0.059	0.554	
After menopaus											
	Log (fasting glucose)				0.028			0.134			0.017
		Sufficiency (≥20 ng/mL)	Reference	Reference		Reference	Reference		Reference	Reference	
		Deficiency (10-<20 ng/mL)	0.0414^*^	0.0008		0.0321	-0.0024		0.0483^*^	0.0023	
		Severe deficiency (<10 ng/mL)	0.0471	0.0014		0.0401	-0.0003		0.0911^*^	0.0140	
		p for trend	0.039	0.761		0.113	0.657		0.003	0.343	
	Log (HbA1c)				0.124			0.258			0.046
		Sufficiency (≥20 ng/mL)	Reference	Reference		Reference	Reference		Reference	Reference	
		Deficiency (10-<20 ng/mL)	0.0200	0.0016		0.0191	-0.0041		0.0149	0.0007	
		Severe deficiency (<10 ng/mL)	0.0252	0.0083		0.0304	0.0080		0.0524^*^	0.0086	
		p for trend	0.148	0.275		0.238	0.911		0.022	0.222	

Values are presented as β coefficients. Adjusted for age, total energy intake, education level, smoking status, obesity, and physical activity. HbA1c, hemoglobin A1c.

* p<0.05,

** p<0.01.

## SUPPLEMENTARY MATERIALS

Supplementary Material 1. Odds ratio for developing diabetes by vitamin D status according to sex and menopause status

Supplementary Material 2. Association between vitamin D and log(HbA1c) by supplemental intake or dietary calcium intake

## References

[1] Holick MF (2007). Vitamin D deficiency. N Engl J Med.

[2] Holick MF (2004). Sunlight and vitamin D for bone health and prevention of autoimmune diseases, cancers, and cardiovascular disease. Am J Clin Nutr.

[3] Hossein-nezhad A, Holick MF (2013). Vitamin D for health: a global perspective. Mayo Clin Proc.

[4] Parker J, Hashmi O, Dutton D, Mavrodaris A, Stranges S, Kandala NB (2010). Levels of vitamin D and cardiometabolic disorders: systematic review and meta-analysis. Maturitas.

[5] Sun Q, Shi L, Rimm EB, Giovannucci EL, Hu FB, Manson JE (2011). Vitamin D intake and risk of cardiovascular disease in US men and women. Am J Clin Nutr.

[6] Giovannucci E (2009). Vitamin D and cancer incidence in the Harvard cohorts. Ann Epidemiol.

[7] May HT, Bair TL, Lappé DL, Anderson JL, Horne BD, Carlquist JF (2010). Association of vitamin D levels with incident depression among a general cardiovascular population. Am Heart J.

[8] Cade C, Norman AW (1987). Rapid normalization/stimulation by 1,25-dihydroxyvitamin D3 of insulin secretion and glucose tolerance in the vitamin D-deficient rat. Endocrinology.

[9] World Health Organization Definition, diagnosis and classification of diabetes mellitus and its complications: report of a WHO consultation. Part 1, diagnosis and classification of diabetes mellitus. https://apps.who.int/iris/handle/10665/66040.

[10] Lim S, Kim DJ, Jeong IK, Son HS, Chung CH, Koh G (2009). A nationwide survey about the current status of glycemic control and complications in diabetic patients in 2006-the Committee of the Korean Diabetes Association on the Epidemiology of Diabetes Mellitus. Korean Diabetes J.

[11] Korea Diabetes Association Diabetes fact sheet in Korea 2018 (ver. English). https://www.diabetes.or.kr/bbs/?code=fact_sheet&mode=view&number=1615&page=1&code=fact_sheet.

[12] International Diabetes Federation (2019). IDF Diabetes Atlas 9th edition. https://diabetesatlas.org/atlas/ninth-edition/.

[13] World Health Organization (2021). Diabetes. https://www.who.int/news-room/fact-sheets/detail/diabetes.

[14] Deshpande AD, Harris-Hayes M, Schootman M (2008). Epidemiology of diabetes and diabetes-related complications. Phys Ther.

[15] Fox CS, Sullivan L, D’Agostino RB Sr, Wilson PW, Framingham Heart Study (2004). The significant effect of diabetes duration on coronary heart disease mortality: the Framingham Heart Study. Diabetes Care.

[16] Banerjee C, Moon YP, Paik MC, Rundek T, Mora-McLaughlin C, Vieira JR (2012). Duration of diabetes and risk of ischemic stroke: the Northern Manhattan Study. Stroke.

[17] Agardh E, Allebeck P, Hallqvist J, Moradi T, Sidorchuk A (2011). Type 2 diabetes incidence and socio-economic position: a systematic review and meta-analysis. Int J Epidemiol.

[18] Hwang J, Shon C (2014). Relationship between socioeconomic status and type 2 diabetes: results from Korea National Health and Nutrition Examination Survey (KNHANES) 2010-2012. BMJ Open.

[19] Choi HS, Kim KA, Lim CY, Rhee SY, Hwang YC, Kim KM (2011). Low serum vitamin D is associated with high risk of diabetes in Korean adults. J Nutr.

[20] Mattila C, Knekt P, Männistö S, Rissanen H, Laaksonen MA, Montonen J (2007). Serum 25-hydroxyvitamin D concentration and subsequent risk of type 2 diabetes. Diabetes Care.

[21] Pittas AG, Harris SS, Stark PC, Dawson-Hughes B (2007). The effects of calcium and vitamin D supplementation on blood glucose and markers of inflammation in nondiabetic adults. Diabetes Care.

[22] Li X, Liu Y, Zheng Y, Wang P, Zhang Y (2018). The effect of vitamin D supplementation on glycemic control in type 2 diabetes patients: a systematic review and meta-analysis. Nutrients.

[23] World Health Organization Regional Office for the Western Pacific (2000). The Asia-Pacific perspective: redefining obesity and its treatment. https://iris.wpro.who.int/handle/10665.1/5379.

[24] Pearce SH, Cheetham TD (2010). Diagnosis and management of vitamin D deficiency. BMJ.

[25] Ozgocmen S, Bulut S, Ilhan N, Gulkesen A, Ardicoglu O, Ozkan Y (2005). Vitamin D deficiency and reduced bone mineral density in multiple sclerosis: effect of ambulatory status and functional capacity. J Bone Miner Metab.

[26] International Diabetes Federation (IDF) (2017). IDF clinical practice recommendations for managing type 2 diabetes in primary care. https://www.idf.org/e-library/guidelines/128-idf-clinical-practice-recommendations-for-managing-type-2-diabetes-in-primary-care.html.

[27] Reichrath J (2007). Vitamin D and the skin: an ancient friend, revisited. Exp Dermatol.

[28] Christakos S, Dhawan P, Liu Y, Peng X, Porta A (2003). New insights into the mechanisms of vitamin D action. J Cell Biochem.

[29] Ishida H, Norman AW (1988). Demonstration of a high affinity receptor for 1,25-dihydroxyvitamin D3 in rat pancreas. Mol Cell Endocrinol.

[30] Fujita T, Palmieri GM (2000). Calcium paradox disease: calcium deficiency prompting secondary hyperparathyroidism and cellular calcium overload. J Bone Miner Metab.

[31] Pittas AG, Lau J, Hu FB, Dawson-Hughes B (2007). The role of vitamin D and calcium in type 2 diabetes. A systematic review and metaanalysis. J Clin Endocrinol Metab.

[32] Reusch JE, Begum N, Sussman KE, Draznin B (1991). Regulation of GLUT-4 phosphorylation by intracellular calcium in adipocytes. Endocrinology.

[33] Teegarden D, Donkin SS (2009). Vitamin D: emerging new roles in insulin sensitivity. Nutr Res Rev.

[34] Sung CC, Liao MT, Lu KC, Wu CC (2012). Role of vitamin D in insulin resistance. J Biomed Biotechnol.

[35] He S, Yu S, Zhou Z, Wang C, Wu Y, Li W (2018). Effect of vitamin D supplementation on fasting plasma glucose, insulin resistance and prevention of type 2 diabetes mellitus in non-diabetics: a systematic review and meta-analysis. Biomed Rep.

[36] Rafiq S, Jeppesen PB (2018). Is hypovitaminosis D related to incidence of type 2 diabetes and high fasting glucose level in healthy subjects: a systematic review and meta-analysis of observational studies. Nutrients.

[37] Mirhosseini N, Vatanparast H, Mazidi M, Kimball SM (2017). The effect of improved serum 25-hydroxyvitamin D status on glycemic control in diabetic patients: a meta-analysis. J Clin Endocrinol Metab.

[38] Santos RK, Brandão-Lima PN, Tete RM, Freire AR, Pires LV (2018). Vitamin D ratio and glycaemic control in individuals with type 2 diabetes mellitus: a systematic review. Diabetes Metab Res Rev.

[39] Nikooyeh B, Neyestani TR, Farvid M, Alavi-Majd H, Houshiarrad A, Kalayi A (2011). Daily consumption of vitamin D- or vitamin D+calcium-fortified yogurt drink improved glycemic control in patients with type 2 diabetes: a randomized clinical trial. Am J Clin Nutr.

[40] Ryu OH, Lee S, Yu J, Choi MG, Yoo HJ, Mantero F (2014). A prospective randomized controlled trial of the effects of vitamin D supplementation on long-term glycemic control in type 2 diabetes mellitus of Korea. Endocr J.

[41] Zoppini G, Galletti A, Targher G, Brangani C, Pichiri I, Negri C (2013). Glycated haemoglobin is inversely related to serum vitamin D levels in type 2 diabetic patients. PLoS One.

[42] Iqbal K, Islam N, Mehboobali N, Asghar A, Iqbal MP (2016). Association of vitamin D deficiency with poor glycaemic control in diabetic patients. J Pak Med Assoc.

[43] Mauvais-Jarvis F, Clegg DJ, Hevener AL (2013). The role of estrogens in control of energy balance and glucose homeostasis. Endocr Rev.

[44] Gupte AA, Pownall HJ, Hamilton DJ (2015). Estrogen: an emerging regulator of insulin action and mitochondrial function. J Diabetes Res.

[45] Korean Diabetes Association Understanding, treatment and management of diabetes. https://diabetes.or.kr/general/info/info_01.php?con=4.

[46] Koo S, Park K (2014). Associations of serum 25 (OH) D levels with depression and depressed condition in Korean adults: results from KNHANES 2008-2010. J Nutr Health.

