# Supplementary material for: Associations of fasting glucose and glycated hemoglobin with vitamin D levels according to diabetes mellitus status in Korean adults
Source: Epidemiol Health. 2022 Feb 21;44:e2022025. doi: 10.4178/epih.e2022025 (PMC9684012; doi:10.4178/epih.e2022025)
Supplement: Supplementary Material 1. — Odds ratio for developing diabetes by vitamin D status according to sex and menopause status [file epih-44-e2022025-suppl1.docx]

| Supplementary Material 1. Odds ratio for developing diabetes by vitamin D status according to sex and menopause status | | | | | | |
| --- | --- | --- | --- | --- | --- | --- |
|  | **Diabetes [n(%)]** | | **OR(95% CI)** | **Diabetes [n(%)]** | | **OR(95% CI)** |
| Vitamin D levels | **No** | **Yes** |  | **No** | **Yes** |  |
|  | **Males (n=14,266)** | |  | **Females (n=18,677)** | |  |
| Sufficient (≥20ng/mL) | 5024(89.71) | 697(10.29) | 1.00 | 4432(91.01) | 507(8.99) | 1.00 |
| Deficiency (10 to <20ng/mL) | 6941(91.72) | 877(8.28) | 1.148(0.990-1.331) | 10891(93.44) | 924(6.56) | 1.029(0.882-1.200) |
| Severe deficiency (<10ng/mL) | 634(90.65) | 93(9.35) | 1.501(1.061-2.123) | 1744(92.56) | 179(7.44) | 1.432(1.120-1.831) |
| p-trend |  |  | 0.011 |  |  | 0.029 |
|  | **Premenopausal (n=9,487)** | |  | **Postmenopausal (n=8,488)** | |  |
| Sufficient (≥20ng/mL) | 1717(97.52) | 46(2.48) | 1.00 | 2555(85.36) | 443(14.64) | 1.00 |
| Deficiency (10 to <20ng/mL) | 6368(97.44) | 169(2.56) | 1.326(0.886-1.985) | 4114(85.36) | 728(14.64) | 0.988(0.836-1.168) |
| Severe deficiency (<10ng/mL) | 1156(97.36) | 31(2.64) | 1.446(0.803-2.603) | 507(79.59) | 141(20.41) | 1.592(1.212-2.089) |
| p-trend |  |  | 0.159 |  |  | 0.034 |
| Adjusted for age, education level, total energy intake, smoking status, physical activity, and obesity. | | | | | | |
