# Supplementary material for: Associations of fasting glucose and glycated hemoglobin with vitamin D levels according to diabetes mellitus status in Korean adults
Source: Epidemiol Health. 2022 Feb 21;44:e2022025. doi: 10.4178/epih.e2022025 (PMC9684012; doi:10.4178/epih.e2022025)
Supplement: Supplementary Material 2. — Association between vitamin D and log(HbA1c) by supplemental intake or dietary calcium intake [file epih-44-e2022025-suppl2.docx]

| **Supplementary Material 2.** **Association between vitamin D and log(HbA1c) by supplemental intake or dietary calcium intake** | | | | | | |
| --- | --- | --- | --- | --- | --- | --- |
|  | **Diabetes** | **Non-diabetes** | **P for interaction** | **Diabetes** | **Non-diabetes** | **P for interaction** |
| **Vitamin D levels** | **β(95% CI)** | **β(95% CI)** |  | **β(95% CI)** | **β(95% CI)** |  |
|  | **Supplement intake group** | | <0.001 | **Non supplement intake group** | | 0.006 |
| **Sufficient (≥20ng/mL)** | Ref | Ref |  | Ref | Ref |  |
| **Deficiency (10 to <20ng/mL)** | 0.0403*  (0.0084-0.0721) | -0.0003  (-0.0050-0.0045) |  | 0.0302  (-0.0007-0.0611) | 0.0011  (-0.0036-0.0058) |  |
| **Severe deficiency (<10ng/mL)** | 0.0669  (-0.0052-0.1389) | -0.0031  (-0.0120-0.0058) |  | 0.0855*  (0.0044-0.1666) | 0.0028  (-0.0050-0.0106) |  |
| **p-trend** | 0.009 | 0.634 |  | 0.014 | 0.481 |  |
|  | **Diabetes** | **Non-diabetes** | **P for interaction** | **Diabetes** | **Non-diabetes** | **P for interaction** |
| **Vitamin D levels** | **β(95% CI)** | **β(95% CI)** |  | **β(95% CI)** | **β(95% CI)** |  |
|  | **High dietary calcium intake group(≥700mg/day)** | | <0.001 | **Low dietary calcium intake group(<700mg/day)** | | <0.001 |
| **Sufficient (≥20ng/mL)** | Ref | Ref |  | Ref | Ref |  |
| **Deficiency (10 to <20ng/mL)** | 0.0444  (-0.0035-0.0924) | -0.0017  (-0.0092-0.0058) |  | 0.0235*  (0.0052-0.0418) | 0.0012  (-0.0025-0.0050) |  |
| **Severe deficiency (<10ng/mL)** | 0.2159**  (0.0791-0.3526) | 0.0029  (-0.0102-0.0161) |  | 0.0333  (-0.0052-0.0719) | 0.0006  (-0.0060-0.0072) |  |
| **p-trend** | 0.002 | 0.999 |  | 0.012 | 0.717 |  |
| **Adjusted for age, sex, education level, total energy intake, smoking status, physical activity, and obesity.** | | | | | | |
